# War on Diabetes in Singapore: a policy analysis

**DOI:** 10.1186/s12961-021-00678-1

**Published:** 2021-02-08

**Authors:** Lai Meng Ow Yong, Ling Wan Pearline Koe

**Affiliations:** Medical Social Services, Singapore General Hospital, Block 3 Level 1, Outram Road, Singapore, 169608 Singapore

**Keywords:** Diabetes mellitus, Health services, Health system, Health policy, Policy analysis, Singapore

## Abstract

**Background:**

In April 2016, the Singapore Ministry of Health (MOH) declared War on Diabetes (WoD) to rally a whole-of-nation effort to reduce diabetes burden in the population. This study aimed to explore how this policy has been positioned to bring about changes to address the growing prevalence of diabetes, and to analyse the policy response and the associated challenges involved.

**Methods:**

This qualitative study, using Walt and Gilson's policy triangle framework, comprised analysis of 171 organizational documents on the WoD, including government press releases, organizational archives, YouTube videos, newspaper reports and opinion editorials. It also involved interviews with 31 policy actors, who were policy elites and societal policy actors.

**Results:**

Findings showed that the WoD policy generated a sense of unity and purpose across most policy actors. Policy actors were cognisant of the thrusts of the policy and have begun to make shifts to align their interests with the government policy. Addressing those with diabetes directly is essential to understanding their needs. Being clear on who the intended targets are and articulating how the policy seeks to support the identified groups will be imperative. Issues of fake news, unclear messaging and lack of regulation of uncertified health providers were other identified problem areas. High innovation, production and marketing costs were major concerns among food and beverage enterprises.

**Conclusion:**

While there was greater public awareness of the need to combat diabetes, continuing dialogues with the various clusters of policy actors on the above issues will be necessary. Addressing the various segments of the policy actors and their challenges in response to the WoD would be critical.

## Background

Diabetes is a condition that affects more than 400 million adults globally, and this number is expected to increase to above 640 million, which equates to one in ten adults, by 2040 [1]. The global prevalence of diabetes among adults over 18 years of age rose from 4.7% in 1980 to 8.5% in 2014 [2]. It was estimated to be the seventh leading cause of death in 2016, where 1.6 million deaths were attributed to the condition [2]. In Singapore, over 400,000 Singaporeans live with the disease. The lifetime risk of developing diabetes is one in three among Singaporeans, and the number of those with diabetes is projected to surpass one million by 2050 [1]. An estimated 430,000 (or 14% of) Singaporeans aged 18 to 19 years are also diagnosed with pre-diabetes, where their normal blood sugar levels are higher than normal but not high enough to be diagnosed as diabetes [3].

In response to this, on 13 April 2016, the Singapore Health Minister declared War on Diabetes (WoD), citing the psychosocial burden on individuals and families and economic reasons for the thrusts of this policy [4]. This fight against diabetes is not new, as Singapore has previously explored measures to combat the rising prevalence of diabetes. For example, the annual National Healthy Lifestyle Campaign, introduced in 1992, aims to raise awareness of how Singaporeans can eat healthier foods and incorporate physical activity into their lives; the campaign concomitantly addresses other concerns such as smoking and mental well-being [5]. Unlike this campaign, the WoD policy specifically addresses the concerns of diabetes and is positioned to encourage a whole-of-society effort to reduce the burden of diabetes in the population and to keep people healthy as they age [1, 3].

Diabetes poses a significant public health concern. It can lead to complications in many parts of the body, including kidney failure, leg amputation, nerve damage, heart attack, stroke, vision loss and severe disabilities [6–8]. It can also bring about substantial economic loss to people and their families and to health systems and national economies as a result of direct medical costs and loss of work and wages [8]. The World Health Organization (WHO) [8], in their 2016 Global Report on Diabetes, calls for a whole-of-government and whole-of-society approach, where all sectors are to systematically consider the health impact of policies in trade, agriculture, finance, transport, education and urban planning. It states that effective approaches, including policies and practices across whole populations and within specific settings, will be needed to contribute to good health for everyone.

This means adopting a life-course perspective and multisectoral and population-based approaches to reduce the prevalence of modifiable diabetes risk factors—such as overweight, obesity, physical inactivity and unhealthy diet—in the general population. It also means addressing the commercial determinants of health, involving multinational or transnational corporations, who are major drivers of noncommunicable disease epidemics, including diabetes, as their strategies and approaches used to promote products and choices could be detrimental to health [9–12].

Since the introduction of the WoD policy, there have been no studies exploring how the policy has been positioned to bring about changes and what the policy actors’ perceived challenges are. Not very much is known about the political, economic, infrastructural and ideational constructivist context in facilitating or hindering the policy at the national and subnational levels [13]. This study thus aims to contribute to addressing this knowledge gap by using the policy triangle framework, articulated by Walt and Gilson [14], to analyse the WoD policy response. The policy triangle framework has been widely applied to a variety of health policy concerns, including health sector reforms and public health, and in many countries [15, 16]. It focuses on the content of the policy, the actors involved in the policy change, the processes in developing and implementing change, and the context within which the policy is developed [14]. The framework is built on the understanding that policy is a product of and constructed through political and social processes [15]. This study will identify the contextual factors that shaped the WoD policy, the actors involved, the content of the policy and organizational provisions, and analyse the strategies and policy processes. Results drawn from this study will be used to inform change agents, such as the relevant government authorities, and will contribute to the body of knowledge on diabetes policy, thereby enhancing the links between science and policy, based on the model of strategic science [17].

## Methods

This study adopted a qualitative approach as the primary method to address the research questions. Qualitative approaches, as opposed to the natural scientific models used in quantitative research, are interpretive and offer an inductive view of the relationship between theory and research [18, 19]. This study comprised interviews with 31 relevant policy actors and members of the general public and the analysis of 171 organizational documents on WoD, including government press releases, organizational archives, YouTube videos, newspaper reports and opinion editorials.

### Participants

We conducted purposive sampling of prospective respondents from five distinct clusters of policy actors, including government officials, healthcare providers, food and beverage (F&B) manufacturers/producers/retailers (small and medium enterprises, or SMEs, to multinational corporations, or MNCs), professional associations, academic institutions/think tanks, and the general public (see Table 1). Non-general public respondents were senior officials within their agencies (for example, president, chief executive officer, general manager, director, deputy director, associate professor) and were actors in or close observers of the WoD policy.

**Table 1:** Respondents by clusters

No.	Cluster	Specific details	Sample size (%)
1	Government officials	Senior officials from Ministry of Health, Health Promotion Board	5 (16.1)
2	Healthcare providers	Endocrinologists, dieticians, medical social workers, diabetes nurse educators	4 (12.9)
3	F&B enterprises	Manufacturers, producers, retailers in the F&B industry	4 (12.9)
4	Professional associations and academic institutions/think tanks	Food manufacturing association, diabetes association, national nutrition and dietetics association, National University of Singapore Saw Swee Hock School of Public Health	4 (12.9)
5	General public	People with and without diabetes, and caregivers of people with diabetes	14 (45.2)
		Total	31

This approach is consistent with the policy triangle analysis framework, where it considers the political institutions and public bureaucracies in policy-making to be important aspects of the analysis. The framework also acknowledges and considers the influence of non-state actors, such as the private sector, the civil society organizations and the public [14, 15]. This is consistent and aligned with WHO’s assertion that non-state actors, such as food producers and manufacturers, healthcare providers and people with diabetes, should be considered collectively in the multicomponent intervention in addressing diabetes [8]. The inclusion of the general public is also relevant because they are driven mostly by their cultural beliefs or personal experiences, which are often the most difficult to identify in terms of their policy goals; their views will therefore be relevant in this policy analysis [20].

### Procedure

All respondents who fulfilled the criteria were invited via letter or email to participate in a semi-structured interview. The interviews were conducted face-to-face in English. Three sets of topic guides comprising semi-structured questions were used for the interviews. They were designed specifically for (a) government officials; (b) healthcare providers, service providers (businesses, food manufacturers, and so on), and professional associations and academic institutions/think tanks; and (c) the general public (with and without diabetes, and caregivers of people with diabetes). The topic guides and interview questions were developed based on the policy triangle framework, articulated by Walt and Gilson [14]. The themes of the topic guides explored participants’ understanding of the following:

- The WoD in terms of its policy goals, impetus, aims and problem definition. Includes who the policy addresses and what the concerns are (context)
- Who the primary players in the policy are (actors)
- The instruments that have been used and parameters that have been put in place, following the introduction of the policy in support of this endeavour (content)
- The key challenges and areas needing to be addressed to better manage the issue of diabetes in Singapore (processes).

As policy and organizational documents constitute the socio-materiality of the policy itself, they were sampled for relevance [21]. All relevant documents within the period 1 January 2016 to 31 December 2019 were reviewed. The documents were obtained directly from the respondents if they were not accessible in the public domain. Documentary analysis was conducted in tandem with face-to-face interviews with the policy actors.

### Data analyses

Data analysis consisted of thematic analysis and analysis of documents, including organizational annual reports, meeting minutes, government press releases (such as government statements; Committee of Supply Speech; speeches for conferences, opening ceremonies, and visits and events by ministers), YouTube videos, newspaper reports and opinion editorials. Thematic analysis was used to analyse data derived from the interviews and documents. The data were read for familiarization and then again in an iterative manner to identify emerging themes. Key categories of codes were analysed and grouped based on the predetermined codes and themes articulated by Walt and Gilson, including context, actors, content and processes [14]. Thereafter, the data derived from both the interviews and documentary analyses were triangulated to enhance the trustworthiness, reliability and validity of the findings [22–24].

## Results

Based on Walt and Gilson’s policy analysis triangle framework, we present the findings below.

### Context

All respondents in this study stated that the reasons for the development and introduction of the WoD policy were numerous. They include the rising prevalence of diabetes, an ageing population, an extended life expectancy, increasing comorbidities of diabetes and rising healthcare costs. In addition, the respondents attributed the introduction of the policy to an increasing economic burden of diabetes on the working population and the associated potential adverse impact on society. These factors together created the moral impetus for the government to introduce the policy to nudge its people into living a healthy lifestyle, respondents stated.

The causes of diabetes were many. Respondents pointed to a complex interaction of economic, social, cultural, individual, national and environmental factors, leading to the formulation of the policy [25, 26]. For example, they highlighted that access to unhealthy food (exacerbated by food delivery service, technology and ready-to-eat meals), affluence of society, expansion of eating-out places, and roles of the F&B industry (manufacturers and retailers) led to the growing diabetes situation in Singapore. This was seen to be made worse by Singaporeans’ obesogenic lifestyle, characterized by work stress, poor sleep patterns and poor overall eating and living habits. The low health screening uptake and lack of prevention measures at the individual level were other reasons. Genetics, invincibility syndrome, culture, family and personal choice, health literacy, and prevailing treatment models of diabetes were seen to have exacerbated the diabetes situation.

### Actors

The actors in the WoD comprised policy elites within the government and societal actors, including the F&B business community (SMEs and MNCs), professional associations, healthcare providers, academic think tanks, civil society and the general public. This policy-led implementation, which is inherently cross-sectoral, saw the Diabetes Prevention and Care Taskforce, set up by the Ministry of Health (MOH), facilitating and coordinating the involvement of the various policy actors. Policy actors such as the F&B business community were quick to acknowledge their corporate and social roles to fellow citizens, and promptly moved to align their business and corporate goals with the policy. Respondent 11, who was from a large MNC fast-food chain, stated:

[A]s cliché as it sounds, it is really a social responsibility on the business part to really care for the customers’ well-being.

The role of the civil society was seen in the involvement of professional associations and voluntary welfare organizations to promote healthier eating and living in the community. Funds were directed to academic and healthcare institutions to encourage and foster diabetes-related research to inform policy and practice. Healthcare institutions were seen to expand their ability to offer better diabetes treatment with increased drug subsidies. Schools, workplaces and organizations implemented policies promoting healthier eating on their premises. The general public were engaged through programmes and schemes, although their level of receptivity and engagement towards the policy varied.

### Content

In operationalizing the policy, a total of 171 WoD-related organizational documents were analysed. The government, in working with the various policy actors and through public forums and engagements, delivered a slew of measures at different time points following the declaration of the policy. The policy core of WoD, highlighted in the documents, centred primarily on increasing the population’s level of physical activity, improving the quality and quantity of dietary intake, increasing early screening uptake and improving intervention to better control diabetes and its associated complications [27].

Notably, in the first 2 years of the policy launch, the government actively used words, images and symbols to form winning coalitions with different policy actors, such as the F&B industry and people with diabetes and their caregivers, and through various languages, including dialects and vernacular languages, to address older adults in the public. The modes of the images included posters, health screening booths and media programmes. Some common symbols and schemes, such as the Healthier Choice Symbol (HCS), Healthier Ingredient Development Scheme (HIDS), Healthier Dining Innovation (HDI), Healthier Dining Grant (HDG) and National Steps Challenges™, targeted consumers, F&B enterprises and the general public.

As part of its overall strategy, the government collaborated with the primary care networks (PCNs) to provide more supportive services for people with diabetes [1]. It subsidized basic screening tests for the public to encourage early detection and treatment. It also put in place systems to foster healthier lifestyles, promote good health by employers in the workplace, and facilitate adjustment of lifestyle habits and better decision-making by individuals [28, 29]. Nonstandard drugs in the treatment of diabetes were subsidized, which helped open up options for primary care physicians to offer newer treatments at lower rates to the general public. According to respondent 5, a physician, older generations of drugs were found to have “potential side-effects and less of non-glucose reducing properties”, whereas “newer drugs have heart failure protection, cardiovascular protection”. This could only benefit patients with diabetes.

The health ministry also partnered with the F&B industry to support major beverage companies and companies undertaking innovation to lower sugar content in their products, by fostering a supportive regulatory environment to encourage innovation and experimentation [30, 31]. This is illustrated in the 2017 industry pact, where seven beverage companies pledged to reduce the sugar level in their beverages to 12% or less by 2020 [32]. This incremental decrease signalled the government’s recognition that innovation and (re)formulation of F&B products would need time, and that immediate introduction of any measures or regulation may backfire. Consumers’ taste acceptance of newer and healthier products would also need time to develop. The MOH further supported and enabled the industry to use Singapore as a regional headquarters and launch pad through which to access other Asian markets to sell their healthier products, to provide the economic conditions for the business community to thrive.

Legal parameters were also explored. A public consultation was carried out from 4 December 2018 to 25 January 2019, where a wide range of stakeholders were engaged for their inputs on introducing mandatory front-of-pack nutrient summary labelling, advertising regulations for the least healthy sugar-sweetened beverages (SSBs), excise duty on manufacturers and importers, and banning of higher-sugar prepackaged SSBs [33]. The proposed measures, which were scheduled to be rolled out later in 2020, came nearly three years after the declaration of the WoD, as the government set the stage to create an environment for its people to lead a healthier lifestyle. In November 2019, the MOH went on to introduce the Patient Empowerment for Self-Care Framework, which constituted the first tranche of materials for people with diabetes to more directly effect change in the lives of those with the condition [34].

### Processes

Several critical factors enabled or constrained the context in the implementation of the WoD. The following discusses the support for and resistance to the WoD policy, and the potential resources that are further needed for its implementation.

#### Why war? Why diabetes?

While the WoD served as a useful “policy frame to galvanize government action, and whole-of-society action and attention”, as stated by a government official (P13), there were considerable competing views among non-policy elites. Many non-policy elite actors, for example, questioned the rationale of the WoD. A member of the general public with diabetes (P19) stated: “I am not sure what the logic is behind using diabetes as the condition, because diabetes is so innocent!” Some respondents, such as P12, a diabetes nurse educator, opined that waging a War on Diabetes was unnecessary, and it might risk perpetuating stigma among those with diabetes. She explained that some of her diabetes patients were upset with the policy and were relatively more withdrawn and “shut off” since its introduction due to their perceived stigma. One of her patients told her, “Then I am not going to tell people I have got diabetes,” because people will relate diabetes to medical complications, she said. Others, including P20, a member of the general public, suggested waging a war against sedentary lifestyle or promoting healthier living might be more appropriate.

Policy actors, particularly professional dieticians and the general public, were unclear whether looking solely at individual nutrients, such as sugar, which was seen to be the primary focus of the WoD, was the best approach to stem diabetes. Respondent 18, a representative from the national nutrition and dietetics association, said: “So I think in a sense we cannot look at individual nutrients; we need to look at diet as a whole. This probably has got to be a very consistent message to the public!” Along the same lines, respondents opined that the policy had focused too heavily on packaged SSBs, rather than on freshly cooked or prepared food. Respondent 3, an MNC F&B manufacturer, highlighted: “The beverage may not be the biggest culprit. In fact, the biggest culprit is food.”

#### Who is the policy for?

Many respondents were unclear of the intended target of the policy. For example, a respondent (P20), a member of the public, reported: “I am not sure who they are targeting, I always thought it is the general public from all age groups.” Another respondent [19], a medical social worker who works with diabetes patients, said: “It is more for the general public, not for those who already have diabetes.” Respondent 29, who has type 1 diabetes, explained: “Type 1 (diabetics) will switch off because it’s like it is too late for them, they already have diabetes.” This sentiment was echoed by respondents with type 2 diabetes and their caregivers, who highlighted that WoD should more directly address their immediate concerns, which would include helping them with their immediate treatment costs and costs of consumables and related devices. For type 1 diabetes, the causal factors were also unclear and it would not be possible to wage war against type 1 diabetes, stated respondent 29. Some respondents observed, and as a government official acknowledged (P13), that pre-diabetic programmes, whilst carefully designed to reduce diabetes incidence, were more accessible to retirees who were available to attend the programmes during workdays, rather than the “supposed” at-risk and younger diabetic groups, who may hold full-time jobs. Others, such as general public respondents P15 and P17, who were both aver 60 years of age, felt that any programme following the policy is good, as it signals a step forward in the fight against diabetes.

#### Messaging quality: unclear images, fake news and diet fads

The barrage of messages pertaining to diabetes was found to be at best overwhelming, at worse conflicting and confusing. Messages such as “white rice is bad” and “too much meat will increase diabetes risk” were confusing to the general public respondents. A respondent (P10), an academic, explained: “Everything you [can't eat] eat also cannot. That’s the flip side of pushing things too hard.” The HCS, which had made significant inroads in encouraging healthier F&B consumption, was found to be unclear in its representation. For example, respondent P10 explained: “If we take drinks with the Healthier Choice Symbol (beverages with lower sugar levels), does it mean drinking five bottles of it will be fine?” Rather than emphasizing a particular nutrient such as sugar, some respondents suggested focusing on individual needs, which might be more appropriate. Fake news and popular commercial “diet fads”, such as the ketogenic and Atkins diets, and intermittent fasting were other concerns reported by respondents. Academic and dietician respondents asserted that consistent advice was lacking, and relevant authorities needed to actively clarify unclear images and fake news, and provide consistent messaging on “diet fads” to the public.

With the proliferation of technology, some professionals and general public respondents highlighted the need to regulate healthcare services provided via online apps and virtual coaching programmes. Respondent 18, a dietician, explained that nutrition coaches on these platforms may not have the necessary qualifications and training, and could in fact, do more harm than good to service users or patients. She asserted that necessary regulation of online healthcare services is crucial to mitigate any potential risks of unregulated online healthcare services.

#### High innovation, production and marketing costs

High innovation, production and marketing costs in the (re)formulation of F&B products were major challenges for the F&B industry respondents. Respondents in this sector explained that taste acceptance for newer and healthier F&B products may not come immediately. F&B retailers, driven by profits, may not be quick to support the sale of healthier products, as the demand for them may not be there at the start. A general manager of an MNC F&B (P3), which produces aerated drinks among other F&B products, highlighted that government support to assist them in engaging in research and development (R&D), marketing, and diversifying and (re)formulating their products would be important and useful. They reported seeing double-digit negative profit margins since the introduction of the policy, and proposed a collaboration that would be beneficial, not just for their corporation, but also for the government and the general public:

We can actually kind of co-create product that we know that is good. Maybe there are certain health concerns, and can do this. Or it could be even at the launch, they [government] could endorse it, or they [government] could give us some promotional funds—how can we jointly, I mean with the help and the support, we can fund it.

Healthier F&B products must also have reach beyond the local market to offset the R&D costs of F&B manufacturers. F&B manufacturer/producer respondents explained that it would mean having to harmonize accreditation of healthier products across countries in order for it to make business sense for them, particularly for a country with a relatively small domestic market like Singapore. To this end, F&B respondents suggested government-to-government and business-to-business collaborations, expressed in forms of shared policies and practices, to give F&B manufacturers the legitimacy to market their (re)formulated healthier products worldwide.

Smaller F&B manufacturers and outlets, such as SMEs, reported acute cash flow issues and were less able to engage in innovation to (re)formulate healthier products. They had to contend with issues such as rising utility costs, rental footprints, high labour costs and limited physical space for stock-keeping units (SKUs) to offer healthier F&B options to their customers. Many respondents questioned the sustainability of rewards, vouchers and subsidies programmes that encourage healthier cooking, eating and living: “Once you finish, then what? I will go back to my own same old way of cooking. I think it’s about sustainability that we need to consider as well before we start on something” (respondent 12, a diabetes nurse educator).

In contrast, F&B retailers, such as larger supermarkets, were least hit by this policy. They were better resourced and better able to offer wider-ranging F&B products with both high and low/no sugar content to their consumers. Larger food establishments, such as restaurants, similarly reported no impact on their profit margins. They were better resourced and were able to offer a wider variety of F&B choices, whether healthier or otherwise, using better-quality and sometimes more expensive ingredients, to meet the needs of consumers who were more willing and able to pay higher prices in these establishments.

## Discussion

This study has explored how the WoD policy has been positioned to bring about changes in its population and the challenges that have arisen as a result. The findings showed that the WoD has generated, to varying extent, a sense of unity and purpose across most policy actors. Policy actors were cognisant of the thrusts of the policy and were quick to make shifts to align their interests with the policy. Legal parameters and economic conditions were debated at public consultations and would be set in place over time. Different policy actors were engaged at various time points. The findings also showed that most respondents demonstrated comprehension and acceptance of the arguments of the policy, and were able to appreciate the implications of diabetes for individuals, institutions and society.

Words, images and symbols were used to strategically shape the policy to produce “winning coalitions” with the policy actors. However, findings showed that there were competing perspectives or views across the policy actors. For example, some non-policy elites wondered whether a war should be waged against diabetes. Specifying diabetes as the target in the WoD could be seen as labelling or blaming those with diabetes and perpetuating stigma via the causal mechanism or action–consequences typology [35]. This causal mechanism has been observed elsewhere and among those with poorer diabetes control or advanced diabetic complications [36, 37]. Sontag [38] cautions that describing disease in terms of siege and war or in the form of “militarized rhetoric” could backfire and may have unintended consequences. There is a need to foster and encourage a positive view towards prevention and treatment of diabetes.

Respondents with diabetes generally did not feel engaged by the policy. Many of them felt that the policy was directed at some “other groups”, but not them. Those with type 1 diabetes, for example, were unsure of who or what the war was being waged against, as the causal factors for type 1 diabetes are unclear. Those with type 2 diabetes reported that the policy should more directly address their underlying concerns regarding treatment costs. Being clear on who the intended targets are and articulating how the policy seeks to help them is important, as it will have implications for the end beneficiaries (winners) and target groups (or losers) [39, 40]. It may also influence the distribution of costs and benefits, as it determines who gets what, when and how, and would have direct implications for practice and implementation [39–41]. Concerns over quality of messaging, information fatigue, diet fads and fake news, and the varying interpretations of the symbols (such as HCS) will need to be addressed.

Mitigating the high innovation, production and marketing costs for policy actors in the F&B industry would be crucial. Larger F&B businesses, including manufacturers, producers, retailers and F&B outlets, which were better resourced and better able to innovate and offer diverse and finer products, reported fewer issues in delivering on the policy. Smaller F&B enterprises—which generally have fewer resources—faced acute cash flow issues related to the necessary innovation and (re)formulation of healthier F&B products. Concerns over sustainability, linkages to marketing agencies, and physical space and costs highlighted the varying interests, paradigms, operational concerns and decision-making processes within the F&B business community and their associated implementation challenges, which will need to be addressed.

It will be crucial to continue to explore the concerns of the F&B industry and to support them in ways specific to their challenges. The individual F&B enterprises may differ in their challenges, depending on where they are situated in the larger business ecosystem and environment. They are also influenced by the nature and range of F&B products they produce or offer, their operational size, and their physical capacity and resources. As many of these business enterprises were quick to acknowledge their corporate and social roles to fellow citizens at the start, it would be imperative that they be supported in this endeavour as the challenges they face are real. Rather than describing their relationship with the government or policy-makers in adversarial terms, and masking them as “conflicts of interest”, it will be important, and perhaps more meaningful, to address their operational challenges head-on, and help them problem-solve to facilitate the implementation of the policy.

Additionally, the role of harmonizing accreditation for healthier products across countries will be critical for the F&B manufacturers, considering the relatively small domestic market in Singapore, to encourage them to engage in R&D for healthier products. A political commitment demonstrated as shared policies by governments to foster innovation and strengthen international partnerships to tackle diabetes and develop healthier F&B products will be crucial [42]. This could be achieved through epistemic communities, policy transfer and policy translation, and collaboration and coordination at the global level.

The role of the F&B enterprises is paramount, and the above discussion has highlighted the importance of making the commercial determinants of health visible. Rather than obscuring the commercial sector responsibility for and contributions to population harms, this study underscores the need to work with these partners to find meaningful ways to work together and ensure policy coherence in tackling the issue of diabetes [43]. Importantly, it also suggests how it may be possible, and in fact necessary, to make certain  that the commercial determinants are consistent with the public interests to positively influence population health. This may mean shifting away from the dominant emphasis in research and policy on clinical management and behavioural change, and towards prevention based on societal and behavioural change [44, 45]. The findings suggest that diabetes should be conceptualized beyond individual-level risk factors, and be reframed as the product of a complex system, in part shaped by the F&B industry [46]. Addressing the various segments of the policy actors and their challenges in response to the WoD is critical. A continued gathering of constant feedback from the various policy actors and exploring ways to support them in this agenda will also be important [47].

### Study strengths and limitations

Current frameworks looking at diabetes prevention and management generally examine the wider determinants of population health, and the commercial or private sector often does not appear to be prominently included [43, 48]. This study explicitly considers their roles and explores how they could be better supported in this WoD to mediate the negative impacts on health arising from their commercial activities. The findings gathered may add to the body of knowledge surrounding commercial determinants of health, where it is still a growing field [12]. The study’s inclusion of those with diabetes, their caregivers and the general public also means that their myriad views are considered and added to the diverse insights into this policy.

All studies have limitations. As with any qualitative research study, the findings cannot be generalized due to its inductive nature. The respective voice of the various policy actors from the five different clusters cannot be generalized, as they each constitute a small number of respondents. Potential respondents who viewed the WoD negatively or were not informed about the policy might not have participated in this study, and their views and experience would not have been reflected. A deep dive to explore the role of social determinants of health on diabetes in the context of the WoD would be useful.

## Conclusion

This study has shown that the WoD policy has generated a general sense of unity and purpose across most policy actors. It has also illustrated the highly complex environment in “doing” policy analysis [49]. The findings showed that the WoD policy needs to segment and engage the clusters of policy actors separately, and to explore their concerns and listen to their voices. In this instance, addressing those with diabetes directly will be critical to understanding their needs, and being clear on who the intended targets are and articulating how the policy seeks to support them is imperative. Issues of fake news, unclear messaging and lack of regulation of uncertified online health providers need to be addressed. High innovation, production and marketing costs should be looked into in greater detail with the F&B enterprises. The policy also needs to be situated at the global stage and environment, to nurture the economic conditions necessary for the F&B industry (manufacturers and innovators in particular) to engage in innovation and venture into (re)formulation of healthier F&B products. Diabetes is a global issue, and efforts to foster and enhance collaboration and coordination across countries on diabetes prevention and management policy is essential and crucial.

## Data Availability

Data can be obtained from the corresponding author on reasonable request.

## Abbreviations

CIRB: Centralised Institutional Review Board
F&B: Food and beverage
HCS: Healthier Choice Symbol
HIDS: Healthier Ingredient Development Scheme
MNCs: Multinational corporations
MOH: Ministry of Health
PCN: Primary care network
SingHealth: Singapore Health Services
SKUs: Stock-keeping units
SMEs: Small and medium enterprises
SSB: Sugar-sweetened beverages
WoD: War on Diabetes
